# Transforming growth factor-β: an important mediator in *Helicobacter pylori*-associated pathogenesis

**DOI:** 10.3389/fcimb.2015.00077

**Published:** 2015-11-04

**Authors:** Nianshuang Li, Chuan Xie, Nong-Hua Lu

**Affiliations:** Department of Gastroenterology, Institute of Digestive Disease, The First Affiliated Hospital of Nanchang UniversityNanchang, China

**Keywords:** *H. pylori*, transforming growth factor-β, gastric inflammation, gastric cancer

## Abstract

*Helicobacter pylori (H.pylori)* is a Gram-negative, microaerophilic, helical bacillus that specifically colonizes the gastric mucosa. The interaction of virulence factors, host genetic factors, and environmental factors contributes to the pathogenesis of *H. pylori*-associated conditions, such as atrophic gastritis and intestinal metaplasia. Infection with *H. pylori* has recently been recognized as the strongest risk factor for gastric cancer. As a pleiotropic cytokine, transforming growth factor (TGF)-β regulates various biological processes, including cell cycle, proliferation, apoptosis, and metastasis. Recent studies have shed new light on the involvement of TGF-β signaling in the pathogenesis of *H. pylori* infection. This review focuses on the potential etiological roles of TGF-β in *H. pylori*-mediated gastric pathogenesis.

## Introduction

Australian scientists Barry Marshall and Robin Warren first identified *Helicobacter pylori (H. pylori)* in 1982 (Marshall, 2008). *H. pylori* is a Gram-negative, microaerophilic, helical bacterium that specifically colonizes the gastric mucosa. More than 50% of people are infected with *H. pylori* worldwide (Eusebi et al., 2014; Graham, 2015), and *H. pylori* infection is strongly associated with chronic gastritis. Additionally, colonization of the stomach with *H. pylori* results in severe gastric diseases, such as intestinal metaplasia, dysplasia, and ultimately gastric carcinoma (Watari et al., 2014). Despite a decreasing incidence of gastric cancer, this disease remains the third leading cause of cancer-related death worldwide (Herrero et al., 2014). Interestingly, infection with *H. pylori* significantly increases the risk of gastric cancer. The International Agency for Research on Cancer (IARC) classifies *H. pylori* infection as a class I carcinogen, and *H. pylori* eradication has been shown to reduce the incidence of gastric cancer (Pan et al., 2015). *H. pylori* infection causes the activation of immune cells, including macrophages, T cells, and B cells, leading to the release of pro-inflammatory cytokines and thus promoting chronic inflammation and the progression to gastric cancer. TGF-β1 not only regulates the initiation and resolution of inflammatory responses but also suppresses immune responses and regulates cancer progression via modulating the expression of multiple genes. The present review discusses the role of TGF-β in *H. pylori*-induced inflammation and the development of gastric carcinoma.

## Pathogenic mechanisms of *H. pylori*

### *H. pylori* virulence factors that influence the gastric epithelium

Several pathogenic mechanisms, including *H. pylori* virulence factors and host factors, have been associated with *H. pylori*-associated gastric diseases. VacA and CagA are major *H. pylori*-secreted proteins that lack known homologs in other bacterial species (Jones et al., 2010). VacA exists in all *H. pylori* strains and encodes vacuolating cell toxins that dysregulate gene expression and other cellular processes (Wada et al., 2010; Palframan et al., 2012). Additionally, VacA causes the apoptosis of gastric epithelial cells through targeting mitochondria and inhibits the proliferation of T cells (Sundrud et al., 2004; Jain et al., 2011). Genetic analysis has suggested that approximately 60% of *H. pylori* strains possess a 40-kb DNA segment known as the *cag* pathogenicity island (PAI), which encodes components of a needle-like type IV secretion system (TFSS) (Hatakeyama, 2014). Cytotoxin-associated gene A (CagA) is transported into the cytoplasm of gastric epithelial cells via the TFSS during *H. pylori* attachment. The presence of CagA-positive *H. pylori* strains increases the risk of peptic ulcers and gastric cancers (Beltrán-Anaya et al., 2014; Song et al., 2014). CagA induces NF-κB activation and the upregulation of proinflammatory immune responses in the host (Lamb and Chen, 2013; Suzuki et al., 2015). Moreover, CagA plays a critical role in gastric carcinogenesis. The CagA protein of *H. pylori* has also been implicated in the Ras-ERK (Yang et al., 2011) and Wnt-beta-catenin signaling pathways that lead to oncogenic mutations (P53, k-ras, etc.; Neal et al., 2013). Other virulence factors of *H. pylori*, such as CagE (Lima et al., 2011), IceA (Boyanova et al., 2010), and BabA (Styer et al., 2010), have also been correlated with gastric diseases. These virulence factors contribute to adherence of and host immune regulation by *H. pylori* within the gastric niche, ultimately resulting in *H. pylori*-mediated gastric inflammation and gastric cancer.

### Host genes involved in the pathogenicity of *H. pylori* infection

In addition to bacterial virulence factors, *H. pylori* infection reprograms host gene expression and modulates various intracellular signaling pathways. Toll-like receptors (TLRs) are central components in innate and adaptive immune recognition. The interaction of *H. pylori* with TLR-signaling pathways also contributes to inflammation. The upregulation of TLRs induces the transcription of molecules in the NF-κB signaling pathway in a MyD88-dependent manner, thereby increasing the levels of inflammatory genes and activating macrophages, which also express the pro-inflammatory cytokines interleukin (IL)-8, IL-1β, and tumor necrosis factor (TNF)-α (Kumar Pachathundikandi et al., 2011; Käbisch et al., 2014). Cyclooxygenase-2 (COX-2) is an enzyme responsible for the pro-inflammatory response (Aoki and Narumiya, 2012). *H. pylori* infection significantly increases the levels of COX2 and prostaglandin E (PGE)-2, thereby contributing to atrophic gastritis and adenocarcinoma (Sierra et al., 2013). Moreover, environmental factors such as smoking and high salt intake are closely linked with *H. pylori* infection (Ghosh and Bodhankar, 2012; Gaddy et al., 2013). Taken together, *H. pylori* bacterial factors, host cell signal transduction, host genetic factors, and environmental factors interact to enhance the mucosal inflammatory response that initiates the multistep process leading to gastric cancer.

## Transforming growth factor-β signaling

### TGF-β superfamily

The multifunctional cytokine TGF-β was discovered in the early 1980s (Garber, 2009). TGF-β regulates cell differentiation, proliferation, wound healing, and angiogenesis via multiple mechanisms. This cytokine also plays an important role in the regulation of tissue homeostasis and the immune system. The TGF-β superfamily includes activins, inhibins, bone morphogenetic proteins (BMPs), growth differentiation factors (GDFS), TGF-β isoforms, and glial cell-derived factors (Shi et al., 2011). TGF-β exists in at least three isoforms: TGF-β1, TGF-β2, and TGF-β3. TGF-β1 is expressed in epithelial, endothelial, and hematopoietic cells; TGF-β2 is expressed in epithelial and neuronal cells; and TGF-β3 is primarily expressed in mesenchymal cells (Papageorgis, 2015). TGF-β1 is stored in a biologically inactive form, containing a signal peptide (SP), latency-associated peptide (LAP), and mature peptide. After intracellular protease digestion, the 25-kDa active TGF-β protein is produced (Horiguchi et al., 2012).

### SMAD and Non-SMAD signaling pathways in TGF-β signaling

TGF-β binds to the type I receptor through TGF-β III and II receptors, resulting in the phosphorylation and activation of TGF-β RI through TGF-β RII in the glycine-serine (GS)-rich domain. The activated TGF-β1 receptor induces Smad2 and Smad3 activation and formation of a SMAD2/3 complex, which in turn interacts with Smad4 and enters the nucleus (Derynck and Zhang, 2003). This SMAD complex recruits co-activators and repressors to regulate the expression of target genes, including the EMT transcription factors snail family zinc finger (SNAIL), twist family bHLH transcription factor 1 (TWIST), zinc finger E-box binding homeobox 1 (ZEB1; Katsuno et al., 2013), matrix metalloproteinases (MMPs; Papageorgis, 2015), plasminogen activator inhibitor 1 (PAI-1; Lang et al., 2014), IL-6, and connective tissue growth factor (CTGF; Reddel et al., 2013).

In addition to SMAD-dependent signaling, the binding of TGF-β to its receptors activates c-Jun N-terminal kinase (JNK), p38 mitogen-activated protein kinase (p38 MAPK; Joko et al., 2013), and external signal-regulated kinase (ERK) signaling pathways (Joko et al., 2013). These SMAD and non-SMAD signaling pathways coordinate to regulate cell proliferation and differentiation.

### Regulation of TGF-β signal transduction

**Figure 1:**
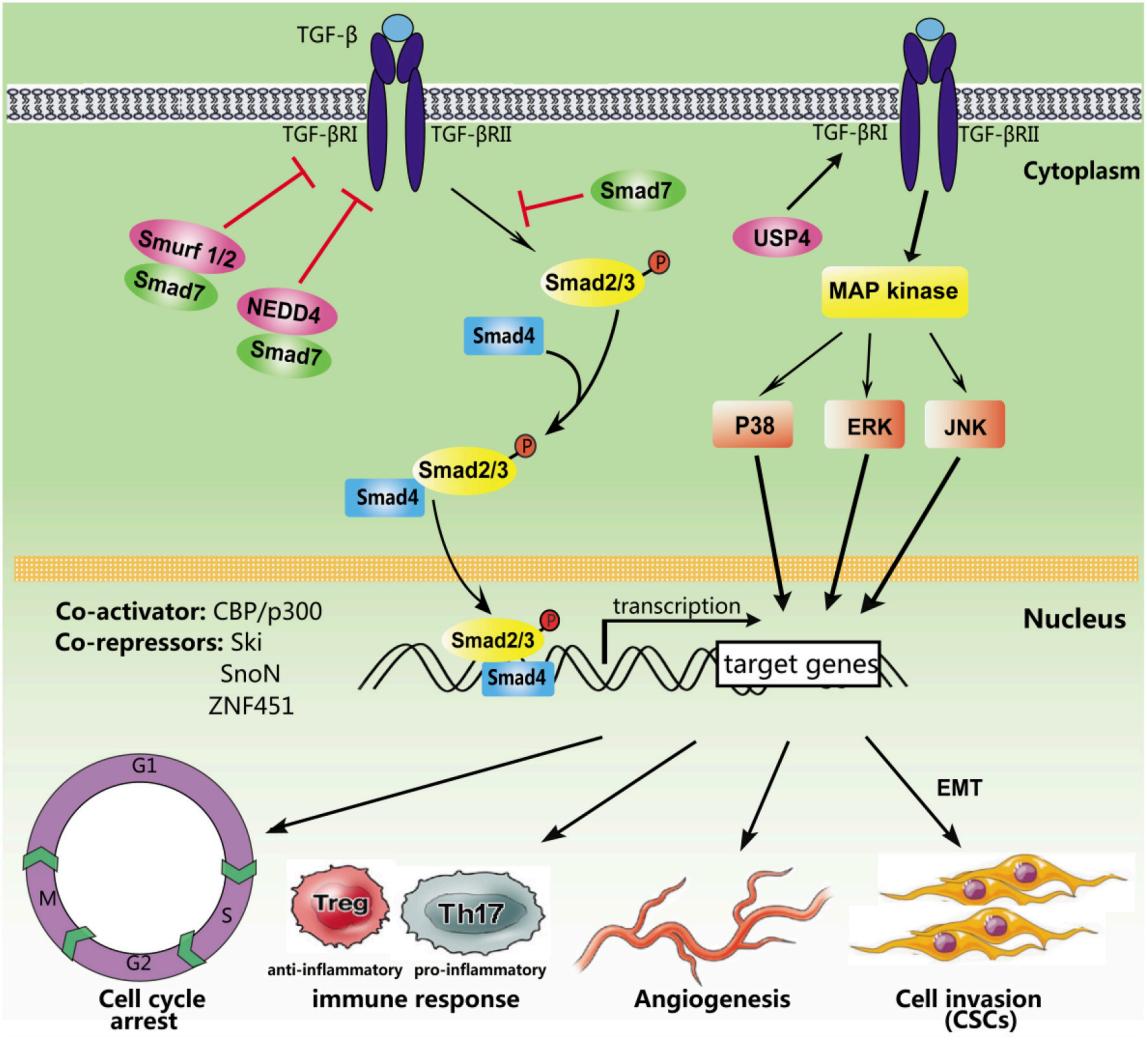
**Simplified TGF-β signaling pathways**. After ligand binding, TGF-β receptors recruit, and phosphorylate intracellular SMAD proteins. Phosphorylated Smad2/3 form a heteromeric complex with SMAD4, which is subsequently transported into the nucleus to regulate the transcription of target genes. Several non-Smad pathways may also be activated. In addition, multiple activators and repressors transcriptionally regulate TGF-β signaling, including CBP/p300, Ski, SnoN, and ZNF451. Smad7 serves as a key antagonist of TGF-β RI by recruiting ubiquitin E3 ligases including NEDD4 and Smurf1/2. However, USP4 could inhibit TGF-β RI degradation. TGF-β signaling regulates different biological processes, such as the cell cycle, the immune response, angiogenesis, and tumor metastasis.

TGF-β primarily evokes cellular responses through SMAD-dependent signaling regulated at the transcriptional level through co-activators and co-repressors (Figure 1). The increased expression of CREB-binding protein (CBP)/E1A-binding protein p300 (CBP/p300) enhances Smad4-dependent transcriptional activation, while zinc finger protein 451 (ZNF451) inhibits the recruitment of p300 through SMAD3/4 complexes in response to TGF-β (Janknecht et al., 1998; Feng et al., 2014). Ski and the closely related SnoN inhibit TGF-β transcriptional responses. SMAD family member 7 (Smad7) negatively regulates the TGF-β/SMAD signaling phosphorylation of R-SMADs (Luo et al., 2014). In addition, Smad7 recruits the HECT-type E3 ubiquitin ligases Smurf1, Smurf2, and NEDD4 (Farooqi et al., 2011), leading to degradation of the targeted protein TGF-β RI. Ubiquitin-specific protease 4 (USP4) augments TGF-β signaling through the prevention of TGF-β RI degradation (Zhang et al., 2012). FAM/USP9x, a deubiquitinating enzyme, controls Smad4 mono-ubiquitination and regulates TGF-β signal transduction (Xie et al., 2014).

### Downstream targets of the TGF-β signaling pathway

Mounting evidence has demonstrated that TGF-β regulates many important cell functions and processes (Figure 1). TGF-β causes cell-cycle arrest in G1 via SMAD-dependent signaling (Yellen et al., 2013) and induces apoptosis through the activation of p38/MAPK signaling (Ferrari et al., 2012). Moreover, TGF-β plays an important role in the regulation of the immune system. CD4+CD25+ regulatory T cells (Tregs) are potent suppressors, maintaining homeostasis and promoting immune tolerance (Facciabene et al., 2012). Previous studies have indicated that TGF-β promotes *Foxp3* gene expression and Treg production (Saini et al., 2014). Furthermore, TGF-β inhibits the activation of lymphocytes and monocyte-derived phagocytes (den Hartog et al., 2013). Activation of the TGF-β/SMAD signaling pathway induces the epithelial-mesenchymal transition (EMT), which initiates and triggers tumor invasion and metastasis (Gao et al., 2014). TGF-β also induces tumor angiogenesis through VEGF-mediated apoptosis (Ferrari et al., 2009). In addition, cancer stem cells are involved in the formation and development of various types of cancers, and recent studies have indicated that TGF-β superfamily members play important roles in the maintenance and differentiation of embryonic (ES) and cancer stem cells (Liu et al., 2015).

## Dysregulation of TGF-β in *H. pylori*-induced host gastric inflammation

### Elevated expression of TGF-β1 and related genes

Host cells recognize pathogen-associated molecular patterns (PAMPs) through pattern-recognition receptors (PRRs), such as TLRs, retinoic acid-inducible gene-I proteins (RIG-Is), and nucleotide oligomerization domain-like receptors (NLRs). PRR-induced signal transduction, including NF-κB signaling, further upregulates the expression of inflammatory factors, ultimately resulting in immune response activation (Jensen and Thomsen, 2012). Previous studies have suggested that TGF-β enhances the attachment to and colonization of host cells by *H. pylori* (Jo et al., 2010), and TGF has been implicated in *H. pylori*-induced gastric mucosal inflammation (Wu et al., 2007), including gastritis and autoimmune disease. As a potentially continuous inflammatory mediator, TGF-β can be induced through a number of cell types, such as macrophages, lymphocytes, and foam cells. The increased expression of TGF-β1 is related to the severity of *H. pylori*-associated non-metaplastic atrophic gastritis (Sun et al., 2009). Previous studies of human gastric mucosal biopsies have revealed that TGF-β1 mRNA expression is significantly increased in *H. pylori*-infected specimens compared with uninfected samples, and this effect is positively correlated with VacA genotype and the grade of chronic inflammation. TGF-β1 is a crucial negative regulator of the immune response through the generation of T-regs. Thus, *H. pylori*-related virulence factor VacA might inhibit T-cell proliferation and immune responses, thereby increasing the adherence of *H. pylori* to the gastric mucosa through the upregulation of TGF-β expression (Rahimian et al., 2014). Serum levels of IL-17A, IL-23, and TGF-β are elevated in patients with *H. pylori* infection, including those with gastritis and peptic ulcers, compared with *H. pylori*-negative populations (Shamsdin et al., 2015), suggesting that *H. pylori*-related inflammation varies depending on the levels of these cytokines. In one study, immunohistochemical staining of proliferating cell nuclear antigen (PCNA) was performed in chronic gastritis patients. Increased immunohistochemical staining of TGF-β, TGF-β RI, and Smad7 was observed in *H. pylori*-positive patients compared with *H. pylori*-negative patients, indicating that the feedback loop incorporating TGF-β1 and Smad7 might play an important role in the progression of *H. pylori* infection (Li and Li, 2006).

### Decreased expression of TGF-β-related genes

**Figure 2:**
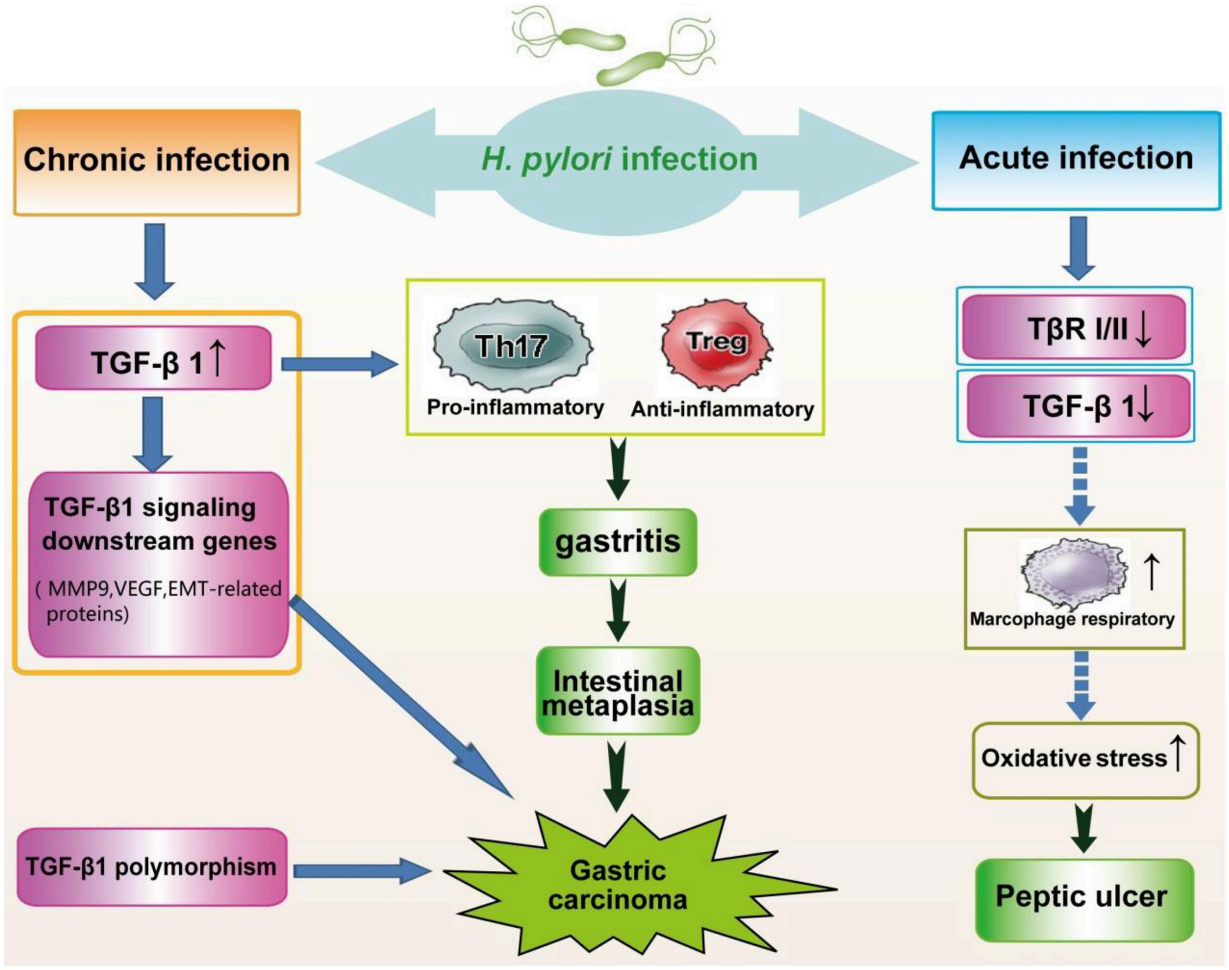
**Alteration of TGF-β signaling in the pathogenesis of *H. pylori* infection**. Chronic infection with *H. pylori* significantly increases the expression of TGF-β1, leading to gastritis and gastric carcinoma. Genes downstream of TGF-β signaling are upregulated in carcinogenesis due to *H. pylori*. In addition, genetic polymorphisms of TGF-β1 are associated with an increased risk of gastric cancer. Conversely, acute *H. pylori* infection, causing peptic ulcer diseases, triggers reduced expression of TGF-β*1*, and TGF-β RI and RII.

Substantially suppressed levels of TGF-β1 have been observed in individuals exposed to *H. pylori* (Figure 2). TGF-β1 levels were markedly decreased in patients with *H. pylori*-associated peptic ulcer diseases. Similarly, *in vitro* studies have shown that the expression of gastric mucosa TGF-β1 is attenuated 24 h post-infection as a host defense mechanism to avoid the attachment of *H. pylori* to gastric epithelial cells. Paradoxically, decreased TGF-β1 expression results in the progression to atrophic gastritis, an autoimmune disease. Because TGF-β1 can suppress the macrophage respiratory burst through the inhibition of H_2_O_2_ release, the downregulation of TGF-β1 contributes to uncontrolled macrophage respiratory burst and severe clinical outcomes associated with oxidative stress (Jo et al., 2010).

In addition, Jo et al. reported markedly lower levels of TGF-β RI and TGF-β RII in patients with *H. pylori*-induced atrophic gastritis compared with an *H. pylori*-negative group (Jo et al., 2010). The significantly increased expression of PCNA following infection suggests that *H. pylori* induces excessive proliferation and apoptosis of gastric epithelial cells, which greatly impairs DNA repair during gastric carcinogenesis. Additionally, TGF-β1-deficient mice exhibit extensive proliferation (García-Sánchez et al., 2010). Therefore, *H. pylori* infection may weaken the inhibitory effect of TGF-β1 on proliferation, leading to substantial hyperplasia of the gastric mucosa through the decreased expression of TGF-β RI and TGF-β RII (Liu et al., 2004).

Smad7 is a well-documented antagonist of the TGF-β signaling pathway. In human gastric adenocarcinoma AGS cells, *H. pylori*-stimulated mononuclear cells (MNCs) in the lamina propria express the TGF-β pathway inhibitory factor Smad7 (Yang et al., 2012).

### TGF-β1 and immune evasion

Virulence factors produced by *H. pylori* provide multiple mechanisms for evading the immune response, thereby causing chronic gastric inflammation. Upon *H. pylori* infection, the gastric mucosa is infiltrated with T cells, including CD4+ T cells (Chrisment et al., 2014). Naive CD4+ T cells differentiate into Th1, Th2, Th17, and Treg subsets (Zhu and Paul, 2010). For example, TGF-β enhances the production of Treg cells through upregulation of the transcription factor Foxp3, which inhibits the activation and proliferation of antigen-specific regulatory T cells, exerting an anti-inflammatory effect (Curotto de Lafaille and Lafaille, 2009). Additionally, CD4+ T cells preferentially differentiate into Th17 cells in response to IL-6 and TGF-β (Beswick et al., 2011; Bailey et al., 2014).

GECs infected with *H. pylori* express highly increased levels of TGF-β1 and TGF-β2, further inducing Foxp3+ Treg cells, which maintain gastritis and facilitate the increased colonization of *H. pylori* through inhibiting the host immune response (Raitala et al., 2007). This effect gradually disappears after knocking out the *H. pylori* virulence factor genes *vacA* and *cagA* (Beswick et al., 2011). However, gastric epithelial cells and monocytes preferentially secrete TGF-β via VacA- and CagA-independent mechanisms (Wu et al., 2007). The expression of TGF-β in patients with peptic ulcers and gastritis is significantly higher than in uninfected counterparts. The positive correlation between the concentration and production of TGF-β from Th17 cells indicates that this cytokine might play a critical role in *H. pylori*-dependent peptic ulcers and gastritis through the regulation of Th17 cells (Shamsdin et al., 2015).

*In vivo*, an *H. pylori*-derived peptide (2–20) stimulates the release of TGF-β and VEGF and induces eosinophil infiltration through interactions with N-formyl peptide receptors (FPRs; Prevete et al., 2013). Immunohistochemical staining of TGF-β in normal gastric mucosa, consistent with its expression in normal fundic mucosa, suggests that this cytokine plays a role in maintaining mucosal homeostasis under physiological conditions (Hawinkels et al., 2007). Overall, TGF-β is a multifunctional cytokine that plays important roles in gastric inflammation through various regulatory mechanisms.

## Dysregulation of TGF-β in *H. pylori*-induced gastric carcinoma

### Alterations of TGF-β signaling in gastric cancer

Alterations of the TGF-β signaling pathway have been observed in the development of gastric cancer. TGF-β1 and TGF-β2 are associated with poor prognosis in gastric cancer. Serum levels of TGF-β1 and TGF-β2 are significantly higher in early and advanced gastric carcinomas compared with control samples (Ma et al., 2013). Furthermore, loss of function mutations in TGF-β RII have been observed in the human gastric cells SNU-5 and SNU-668, which are resistant to growth inhibition by TGF-β. Reintroduction of the TGF-β RII gene reversed TGF-β-induced tumorigenicity and clonogenicity in these cells. Overall, TGF-β RII is a potential tumor suppressor gene in gastric cancer cells (Yang et al., 1999; Takeno et al., 2002). The hypermethylation of CpG islands in the TGF-β RI promoter region has been well-documented in sporadic gastric carcinomas. TGF-β-resistant T cells treated with a demethylating agent and transiently transfected with TGF-β RI demonstrated restored TGF-β responsiveness (Kubiczkova et al., 2012).

Moreover, TGF-β1 promotes the invasion and metastasis of gastric cancer cells through the induction of Fascin 1, an actin-binding protein. Treatment with MAPK pathway-specific inhibitors, in turn, reverses these biological activities (Fu et al., 2009). Furthermore, *in vitro* studies have indicated that TGF-β1 induces apoptosis through TGF-β receptors I and II and a p53-independent pathway (Yamamoto et al., 1996).

Impaired SMAD proteins associated with the TGF-β signaling pathway have also been detected in gastric carcinoma. Phosphorylated-Smad2 is a key intracellular molecule for TGF-β signal transduction. The immunohistochemical expression of p-Smad2 was determined in advanced gastric adenocarcinomas from 135 patients and found to be significantly higher in diffuse type carcinomas, tumors with peritoneal metastasis, and tumors with lymph node metastasis, implying that activated Smad2 might be positively correlated with malignant gastric cancer (Shinto et al., 2010). In one study, the expression of the common SMAD mediator Smad4 and the inhibitory SMAD protein Smad7 was examined in gastric adenocarcinomas. *Smad4* gene expression was lacking, and this loss was associated with the depth of tumor invasion and poor survival. However, Smad7 expression in well-differentiated gastric adenocarcinomas was significantly higher than that in the normal gastric mucosa and associated with the duration of disease-free survival (Zizi-Sermpetzoglou et al., 2014).

### Genetic polymorphisms of TGF-β1 and gastric cancer

Recent studies have shown that C-509T gene polymorphisms in the promoter region of TGF-β1 are associated with plasma levels (Hosseini Razavi et al., 2014). Similarly, the TGF-β1 T869C polymorphism affects the secretion of TGF-β1, suggesting that the polymorphic variants of TGF-β1 might influence cancer risk (Peng et al., 2011). In Chinese populations, the C-509T and T29C polymorphisms are correlated with decreased gastric cancer risk among stage I+II cases and increased risk for stage III+IV gastric cancers (Zhang et al., 2008). Moreover, the TGF-β1 T869C gene polymorphism has been implicated in susceptibility to *H. pylori*-related diseases (Garcia-Gonzalez et al., 2006). Clinical observations have shown that CagA-positive patients with the TGF-β1 promoter polymorphism C-509T are at increased risk for *H. pylori*-associated gastric precancerous lesions (Achyut et al., 2009). Accordingly, TGF-β1 polymorphisms may be a susceptibility factor for the occurrence and development of gastric cancer (Figure 2).

### Alteration of the TGF-β signaling pathway in *H. pylori*-induced gastric carcinoma

Infection with *H. pylori* is a strong risk factor for gastric cancer, and most gastric cancer cases are attributable to *H. pylori* infection. Transforming growth factor-β has been implicated in various biological processes, including cell cycle regulation, apoptosis, tumor angiogenesis, tumor invasion, and cancer cell metastasis. Many advanced tumors, such as those of the stomach and breast, show excessive expression of TGF-β. The methylation of TGF-β RI, TGF-β RII, and Smad4 has been observed during the early stages of gastric adenocarcinoma (Guo et al., 2012). In addition, elevated TGF-β1 and IL-10 serum levels in gastric cancer patients infected with *H. pylori* have also been observed (Szkaradkiewicz et al., 2010). Transgenic mice expressing a dominant-negative mutant of TGF-β RII show a loss of TGF-β signaling, particularly in the stomach, promoting cell proliferation and higher incidences of gastrointestinal cancers (Hahm et al., 2002a). Dequchi et al. reported a TGF-β RII gene mutation in CagA-positive *H. pylori*-infected patients (Deguchi et al., 2001). In addition, the TGF-β1 promoter is methylated in gastric cancer patients, and the levels of TGF-β1 methylation in *H. pylori*-positive gastric mucosal tissues are significantly higher than those in *H. pylori*-negative gastric mucosal tissues (Wang et al., 2013).

Alterations of the TGF-β signaling pathway have been observed in the development of gastric cancer. The epithelial-mesenchymal transition (EMT) induces the invasion and metastasis of *H. pylori*-associated gastric cancer and the emergence of cancer stem cells (CSCs), in which epithelial cells lose cell polarity and cell-cell adhesion, subsequently breaking through the basement membrane and metastasizing to distant sites (Tsai and Yang, 2013; Yu et al., 2014). TGF-β induces EMT through a SMAD-dependent pathway. In AGS and MKN45 gastric cancer cells, CagE-positive *H. pylori* infection promotes the expression of EMT-related markers, enhancing cell invasion and migration. Transient transfection with the G27 CagE mutant reverses these protein levels and induces pathophysiological changes in cell morphology (Chang et al., 2015). *In vivo* studies have shown the upregulation of TGF-β1 and EMT-related genes in dysplasia and early gastric cancer patients infected with *H. pylori*. A significant reduction in the mRNA levels of EMT markers has been observed after *H. pylori* eradication. CD44, a well-known marker for CSCs, shows increased expression in *H. pylori*-positive gastric carcinomas (Choi et al., 2015). Gastric epithelial cells co-cultured with a CagA-positive *H. pylori* strains or transfected with CagA expression vectors also induce EMT-related mesenchymal markers and exhibit increased tumorigenic properties (Bessède et al., 2014; Lee et al., 2014). In summary, TGF-β/EMT signaling plays a critical role in *H. pylori*-induced carcinogenesis.

In addition, alterations in effectors downstream of the TGF-β signaling pathway have been observed in the carcinogenesis of *H. pylori* infection (Figure 2). Gastric carcinoma tissues positive for the L-form of *H. pylori* (*H. pylori*-L) show significantly increased MMP-9 and VEGF expression, which are regulated through the TGF-β signaling pathway (Ou et al., 2014). Additionally, the signaling pathways of BMPs, which are additional members of the TGF-β superfamily, have been implicated in the pathogenic stages of gastric cancer, including intestinal metaplasia, and gastric cancer associated with *H. pylori* (Bleuming et al., 2006; Camilo et al., 2012).

*H. pylori* infection contributes to the progression of superficial gastritis to atrophic and intestinal metaplasia, ultimately leading to gastric cancer. Th17 cells, a lineage of CD4+ T cells, promote tumor growth. *H. pylori*-infected human gastric cancer tissues co-cultured with CD4+ T cells induce the production of Th17 cells via TGF-β and IL-6 secretion (Pinchuk et al., 2013).

COX2 plays a key role in *H. pylori*-induced gastric carcinoma. In one study, the stable transfection of COX2 into MKN-45 and MKN-28 cells attenuated NF-κB signaling. Additionally, *H. pylori* infection decreased the expression of TGF-β RII and evaded growth inhibition through TGF-β, thereby facilitating cell invasion (Hahm et al., 2002b).

## Conclusions and perspectives

In this review, we summarized previous studies of the multiple effects of TGF-β in the pathogenesis of *H. pylori* infection. The dysregulation of TGF-β and related cytokines is highly widespread in *H. pylori*-related diseases. However, current evidence is limited to comparative studies of TGF-β-related gene expression and functional studies in different *H. pylori*-infected human tissues. To our knowledge, the TGF-β signaling pathway, including SMAD and non-SMAD pathways, plays a vital role in cancer formation, progression and metastasis. Therefore, understanding the mechanisms underlying the multiple roles of the TGF-β signaling pathway in pathologies associated with *H. pylori* infection, particularly gastric carcinogenesis, will provide valuable information for future studies.

### Conflict of interest statement

The authors declare that the research was conducted in the absence of any commercial or financial relationships that could be construed as a potential conflict of interest.
